# Challenges in Implementing Endoscopic Artificial Intelligence: The Impact of Real‐World Imaging Conditions on Barrett's Neoplasia Detection

**DOI:** 10.1002/ueg2.12760

**Published:** 2025-03-21

**Authors:** M. R. Jong, T. J. M. Jaspers, C. H. J. Kusters, J. B. Jukema, R. A. H. van Eijck van Heslinga, K. N. Fockens, T. G. W. Boers, L. S. Visser, J. A. van der Putten, F. van der Sommen, P. H. de With, A. J. de Groof, J. J. Bergman, The BONS‐AI consortium

**Affiliations:** ^1^ Department of Gastroenterology and Hepatology Amsterdam Gastroenterology, Endocrinology and Metabolism Amsterdam UMC University of Amsterdam Amsterdam The Netherlands; ^2^ Department of Electrical Engineering Eindhoven University of Technology Eindhoven The Netherlands

**Keywords:** artificial intelligence, Barrett's esophagus, computer aided detection, deep learning systems, endoscopy, esophageal adenocarcinoma

## Abstract

**Background:**

Endoscopic deep learning systems are often developed using high‐quality imagery obtained from expert centers. Therefore, they may underperform in community hospitals where image quality is more heterogeneous.

**Objective:**

This study aimed to quantify the performance degradation of a computer aided detection system for Barrett's neoplasia, trained on expert images, when exposed to more heterogeneous imaging conditions representative of daily clinical practice. Further, we evaluated strategies to mitigate this performance loss.

**Methods:**

We developed a computer aided detection system using 1011 high‐quality, expert‐acquired images from 373 Barrett's patients. We assessed its performance on high, moderate and low image quality test sets, each containing images from an independent group of 117 Barrett's patients. These test sets reflected the varied image quality of routine patient care and contained artefacts such as insufficient mucosal cleaning and inadequate esophageal expansion. We then applied three methods to improve the algorithm's robustness to data heterogeneity: inclusion of more diverse training data, domain‐specific pretraining and architectural optimization.

**Results:**

The computer aided detection system, when trained exclusively on high‐quality data, achieved area under the curve (AUC), sensitivity and specificity scores of 83%, 85% and 67% on the high quality test set. AUC and sensitivity were significantly lower with 80% (*p* < 0.001) and 62% (*p* = 0.002) on the moderate‐quality and 71% (*p* > 0.001) and 47% (*p* = 0.002) on the low‐quality test set. Incorporating robustness‐enhancing strategies significantly improved the AUC, sensitivity and specificity to 92% (*p* = 0.004), 88% (*p* = 0.84) and 81% (*p* = 0.003) on the high‐quality test set, 93% (*p* = 0.006), 86% (*p* = 0.01) and 83% (*p* = 0.09) on the moderate‐quality test set and 84% (*p* = 0.001), 78% (*p* = 0.002) and 77% (*p* = 0.23) on the low‐quality test set.

**Conclusion:**

Endoscopic deep learning systems trained solely on high‐quality images may not perform well when exposed to heterogeneous imagery, as found in routine practice. Robustness‐enhancing training strategies can increase the likelihood of successful clinical implementation.

## Graphical abstract

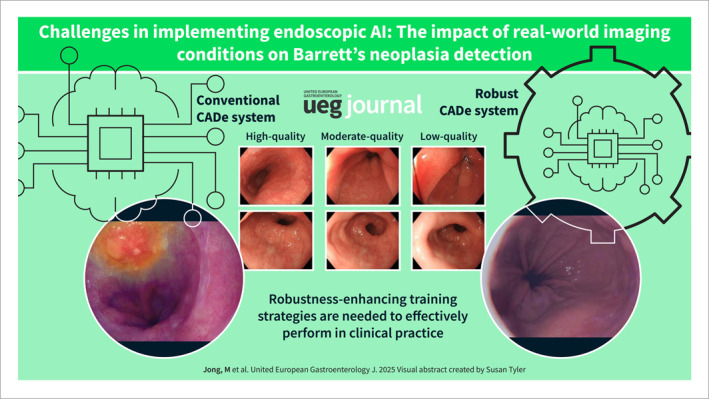

1

**Summary**

- Summarize the established knowledge on this subject
  ◦ Current endoscopic AI systems are predominantly trained on high‐quality, expert‐acquired datasets that do not reflect the diverse imaging conditions encountered in routine clinical practice. This mismatch leads to a phenomenon known as the “domain gap.”
  ◦ There is limited data regarding the impact of this domain gap on the performance of AI systems.
- What are the significant and/or new findings of this study?
  ◦ This study is the first to comprehensively evaluate the performance decline of endoscopic AI systems when exposed to heterogeneous input data, using Barrett's neoplasia detection as an illustrative example.
  ◦ Performance of endoscopic AI systems, trained on high‐quality data, decreases significantly when exposed to varied imaging conditions, posing a major challenge for successful clinical implementation.
  ◦ Future endoscopic AI systems should prioritize robustness to data heterogeneity during both algorithm development and performance evaluation.

## 2 Introduction

Over the past decade, the medical domain has witnessed a surge of artificial intelligence (AI) systems [1]. This also holds true for the field of gastrointestinal endoscopy [2, 3]. Several of these AI applications have received FDA approval [4]. However, it is not uncommon that AI systems underperform in clinical practice compared with their original efficacy in controlled studies [5, 6, 7]. A core underlying issue is that virtually all endoscopic AI systems have been developed and evaluated in tertiary academic centers, providing ideal conditions with high‐quality datasets acquired by experts. In contrast to other imaging modalities, such as CT or MRI, the image quality in endoscopy relies heavily on the endoscopist who performs the procedure. In community‐based centers, the input data for an AI algorithm can be significantly more heterogeneous. This creates a so‐called “domain gap,” a common phenomenon where AI displays excellent performance on familiar data and a converse drop when confronted with data that differs from its training distribution [8, 9]. For truly meaningful impact, AI systems should produce robust and reliable results, especially in community hospitals where the majority of these systems will be employed.

**FIGURE 1:**
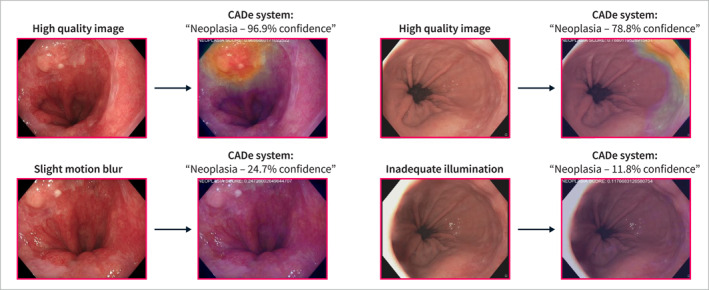
Example cases displaying the consequences of minimal image quality variation. The CADe system suffers from significant performance loss when confronted with lower quality images of the same patient.

Recently, our group developed and validated a computer aided detection (CADe) system for Barrett's neoplasia [10, 11, 12]. Despite the unparalleled volume and heterogeneity of the datasets as well as a rigorous evaluation process, all data originated from expert centers. CADe performance may become less reliable when the system is confronted with data with suboptimal image quality (Figure 1). Strategies to improve the *robustness* of CADe systems against a wide variety of endoscopic image quality are warranted.

While the evaluation of robustness in computer vision is a well‐known topic, it remains a largely unexplored area in the field of endoscopy. A previous study by our group [13] displayed a significant lack of robustness of endoscopic AI systems on datasets when exposed to artificially degraded images. In this study, we aimed to quantify the expected decline in CADe performance when transitioning from high‐quality imagery to a broader spectrum of image quality, as may be encountered in daily practice. In addition, we evaluated preliminary strategies to reduce performance decline.

## 3 Methods

### 3.1 Experimental Set‐Up

**FIGURE 2:**
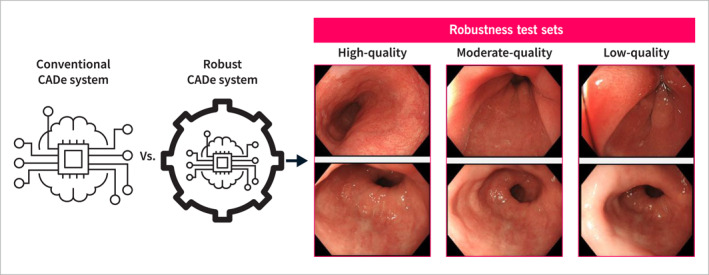
Comparison of a conventionally trained CADe system with a robust CADe system across three test sets comprising the complete spectrum of image quality.

For the purpose of our experiments, we first developed a new CADe system for Barrett's neoplasia along the lines of our previous work [10, 12]. This *conventionally trained* system was trained solely on high‐quality expert‐acquired imagery. We then constructed a test set that included images spanning the full range of endoscopic image quality. This variety represented the potential input that a CADe system might encounter, from dedicated high quality images taken by expert endoscopists in tertiary centers to lower quality images encountered in daily practice. This robustness test set was divided into three subsets: high‐, moderate‐, and low‐quality images. The performance of this conventionally trained CADe system was evaluated on the high quality test set and subsequently on the moderate and low‐quality test sets to quantify the domain gap. We then developed a more robust CADe system using three robustness enhancing training methods (described below). We evaluated the performance of this more robust CADe system on the same three test sets and compared its performance with the baseline performance of the conventionally trained CADe system. For secondary analyses, we trained three additional CADe systems based on only one robustness enhancing method. The experimental set‐up is displayed in Figure 2.

### 3.2 Data Acquisition

This study was conducted by the BONS‐AI consortium (Barrett's esophagus imaging for Artificial Intelligence). All data were collected in a strictly anonymized manner and originated from previous studies [10, 12], which were registered at the Dutch Trial Register under the number NL8411. The participating BONS‐AI centers collected prospective imagery using a standardized protocol for image and video acquisition. This protocol has been described in an earlier publication [10].

### 3.3 Conventionally Trained CADe System

The conventionally trained CADe system was designed reflecting the developmental approach of most currently used endoscopic AI systems. The training dataset consisted exclusively of dedicated still images acquired by expert endoscopists from 15 international tertiary centers according to a standardized protocol [10], using HQ190/EZ1500 endoscopes with CV190/X1 processors (Olympus, Tokyo). Images were captured under optimal conditions, including extensive mucosal cleaning, adequate esophageal expansion, and consistent lighting, ensuring high‐quality standards. It comprised 538 images from 184 neoplastic patients and 564 images from 206 non‐dysplastic Barrett's esophagus patients. To rigorously assess its performance, 5‐fold cross‐validation was conducted with a patient split. Given its frequent use and wide acceptance for medical image classification tasks, the ResNet‐50 architecture was selected as the foundation for the CADe systems used in this study. Training parameters such as learning rate, number of epochs and data augmentation were kept fixed for all experiments and chosen based on experimental findings for all models to converge. More specific details on data acquisition, curation and model hyperparameter selection are described in the Supporting Information S1: (p. 3).

### 3.4 Robust CADe System

The CADe system was then updated using three robustness enhancing training methods. These were all integrated into one *robust* CADe system aimed to be more resistant to varying levels of image quality.

#### 3.4.1 Inclusion Diverse Training Data

The most intuitive solution to improve the robustness of CADe systems is to incorporate a wider variety of endoscopic image quality into the training set. Ideally, images originating from non‐expert centers should be used for this purpose, but due to the low prevalence of Barrett's neoplasia in these hospitals, this is practically not feasible. As an alternative, we elected to include video frames. In contrast to still images (where the endoscopists makes a decision call to store or to discard the image), videos contain a wider variety of image quality, even in expert‐acquired videos. We randomly sampled video frames from curated neoplastic and non‐dysplastic sequences from the same patient cohort used for the conventional CADe system with identical endoscopy equipment. These video sequences were selected to contain a wide variety of endoscopic image quality, but were strictly curated to make sure that neoplastic sequences did indeed comprise visual neoplasia on every frame. Image quality varied across factors such as mucosal cleanliness, esophageal expansion, lighting, and motion artifacts. The dataset generated with this process thereby represented imagery that can be expected to be found in a non‐expert setting. It contained 5870 video frames from 184 neoplastic patients and 6141 video frames from 206 non‐dysplastic Barrett's esophagus patients. Examples of this video frame training set are given in Supporting Information S1: Figure S1.

#### 3.4.2 Domain‐Specific Pretraining

Traditionally, before actual training on application‐specific data (e.g., images of Barrett's neoplasia or colorectal polyps), computer vision algorithms are generally *pre‐trained* on large, publicly available datasets such as ImageNet [14], containing generic images (e.g., animals, vehicles and buildings). From these images, an algorithm can learn to recognize basic image characteristics such as colors, edges and simple shapes. This is a valuable and essential step in algorithm development, especially for tasks where training data is limited, such as in the medical domain. Studies suggest that utilizing large datasets of *in‐domain* images, that is, endoscopic images, for pretraining results in improved algorithm performance [15, 16]. We recently proposed GastroNet‐5M [17, 18], a dataset of over 5 million unlabeled general endoscopic images intended for *domain‐specific pretraining*. In preliminary experiments, the use of GastroNet‐5M improved algorithm robustness against artificially generated endoscopic quality artifacts such as a blurry lens, motion blur and inadequate illumination [13]. In this study, we substituted ImageNet with GastroNet‐5M as one of the three robustness enhancing training methods.

#### 3.4.3 Algorithm Architecture

Currently, most AI applications in endoscopy are based on so called convolutional neural networks (CNNs). Therefore, we used the commonly used and widely accepted ResNet‐50 architecture, a CNN, for our conventionally trained CADe system. In 2021, vision transformers (ViTs) were introduced as a powerful alternative to CNNs [19]. This architecture has enabled significant improvements in the field of medical imaging [20]. Our group has presented empirical evidence suggesting its potential to improve endoscopic AI applications in terms of performance and robustness to data variability [21]. Therefore, we implemented an updated algorithm architecture mostly based on the ViT architecture but also incorporating some components of CNNs [22].

All robustness enhancing training methods have been summarized in Table 1.

**TABLE 1:** Differences between the conventionally trained and robust CADe systems.

Aspect	Conventional CADe system	Robust CADe system
Training data type	Still images	Video frames
Training data size	538 neoplastic images (184 patients); 564 non‐dysplastic images (206 patients)	5870 neoplastic frames (184 patients); 6141 non‐dysplastic frames (206 patients)
Training data quality	High‐quality only (clean mucosa, adequate esophageal expansion, clear images)	Diverse quality (varying degrees of mucosal cleaning and esophageal expansion; may contain blur and illumination artefacts)
Pretraining	Generic (ImageNet‐1K)	Domain‐specific (GastroNet‐5M)
Model architecture	Convolutional neural network (CNN)	Hybrid CNN‐Transformer

### 3.5 Robustness Test Sets

**FIGURE 3:**
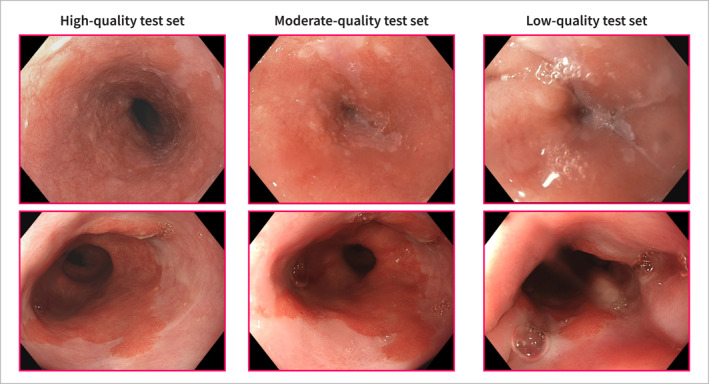
Representative neoplasia cases from three different quality test sets: high‐quality (left), moderate‐quality (center), and low‐quality (right).

The *robustness test sets* aimed to represent the varying levels of endoscopic image quality that may be encountered in clinical practice. These consisted of three subsets based on three different levels of image quality: a *high‐, moderate‐, and low‐quality test set*. All subsets were paired on a per patient basis. Endoscopic image quality can be affected by several factors. In this study, we focused on *endoscopist dependent* image quality parameters such as esophageal expansion, lighting conditions, blurriness and degree of esophageal cleaning (Supporting Information S1: Figure S2). The test sets were constructed by a manual selection of video frames from an independent patient population of 61 neoplastic and 56 non‐dysplastic patients which were not included in the training sets. For each patient, a matched triplet of video frames was collected by a research fellow (MRJ), and this selection was subsequently confirmed by an expert Barrett's endoscopist (JJB). This triplet comprised a high‐quality, a medium‐quality and a low‐quality video frame (Figure 3) of the same patient and the same position in the Barrett's segment. Further test set specifications are listed in Supporting Information S1: Table S1.

### 3.6 Outcome Measures

We identified two *primary outcome measures* for the conventionally trained CADe and our robust CADe system: (1) *Absolute performance,* represented by the Area‐under‐the‐Curve (AUC), sensitivity and specificity scores on the high, moderate and low‐quality test sets, and (2) *Performance difference,* defined as the difference in AUC, sensitivity and specificity scores on the moderate and low‐quality test set compared to the high‐quality test set. *Secondary outcome measures* were absolute performance and performance difference from the CADe systems encompassing solely one of the three robustness enhancing methods (i.e., incorporation of video frames, domain‐specific pretraining or the ViT architecture).

### 3.7 Statistical Analysis

The conventionally trained CADe system functioned as a baseline for all subsequent CADe systems trained with experimental robustness improvement methods. For every CADe system, we performed 5‐fold cross‐validation and reported the means. These five folds allowed for direct comparison of CADe systems using two‐sided paired t‐tests. Direct comparisons were made for both outcome measures: (1) absolute performance, comparing different CADe systems on the same test sets and (2) performance difference, comparing the same CADe system on different test sets. In all instances, a *p*‐value of less than 0.05 was considered statistically significant. All calculations were conducted utilizing Python 3.8 (Python Software Foundation).

## 4 Results

### 4.1 Primary Outcome Measures

#### 4.1.1 Conventionally Trained CADe System

On the high‐quality test set, the conventionally trained CADe system reached an AUC score of 83%. On the moderate‐quality test set, AUC decreased significantly to 80% (*p* = 0.0007). AUC score on the low‐quality test set decreased even further to 71% (*p* < 0.0001). This loss could mainly be attributed to a loss of sensitivity, which was 85% on the high‐quality test set and decreased to 62% and 47% on the moderate‐ and low‐quality test sets, respectively.

#### 4.1.2 Robust CADe System

Compared to the performance of the conventionally trained CADe system, the robust CADe system reached a higher AUC on the high‐, moderate‐ and low‐quality test sets with scores of 92% (*p* = 0.004), 93% (*p* = 0.0006) and 85% (*p* = 0.0001). In addition, the difference in the AUC score of the robust CADe system on the moderate and low‐quality test sets was significantly smaller (*p* = 0.004 and *p* = 0.01), when compared to the conventionally trained system. This reduction in performance loss was primarily due to the limited sensitivity loss, which decreased from 88% on the high‐quality test set to 87% and 78% on the moderate‐ and low‐quality test sets.

**FIGURE 4:**
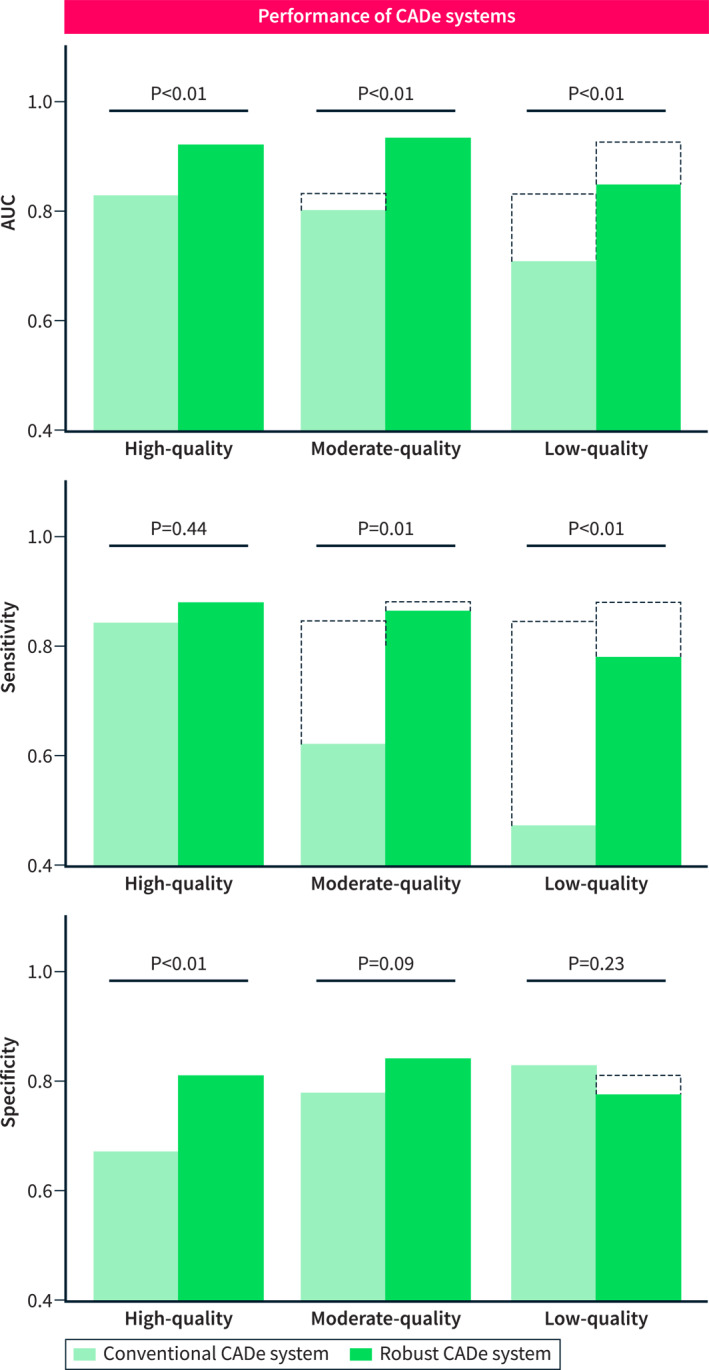
Results of the conventionally trained CADe system versus the robust CADe system. Dashed bars represent the scores on the high‐quality test set.

Results are summarized in Figure 4 and Table 2.

**TABLE 2:** Results of both CADe systems on the high, moderate and low‐quality test sets.

Test set	Metric	Conventional CADe system	Robust CADe system	*p*‐value
High‐quality	AUC	83%	92%	0.0039
	Sensitivity	85%	88%	0.4415
	Specificity	67%	81%	0.0025
Moderate‐quality	AUC	80%	93%	0.0006
	Sensitivity	62%	87%	0.0108
	Specificity	78%	84%	0.0865
Low‐quality	AUC	71%	85%	0.0001
	Sensitivity	47%	78%	0.0024
	Specificity	83%	77%	0.2343

### 4.2 Secondary Outcome Measures

We then evaluated the contributions of each individual robustness‐enhancing method.

#### 4.2.1 Diverse Training Data (Video Frames)

The baseline CADe system updated with a training set comprising video frames of more diverse quality reached higher AUC scores compared to the conventionally trained CADe system on the high‐quality (87%; *p* = 0.05), moderate‐quality (85%; *p* = 0.01) and low‐quality (76%; *p* = 0.17) test sets. The corresponding AUC difference in moderate (−1%; *p* = 0.04) and low‐quality (−11%; *p* = 0.63) test sets was reduced.

#### 4.2.2 Domain‐Specific Pretraining

After incorporating the GastroNet‐5M dataset for domain‐specific pretraining into the baseline CADe model, the GastroNet‐based CADe system reached higher AUC scores on the high, moderate and low‐quality test sets with 95% (*p* = 0.0004), 89% (*p* = 0.007) and 76% (*p* = 0.14), respectively. Despite this, AUC loss differences were actually larger on the moderate (−5%; *p* = 0.11) and low‐quality (−19%; *p* = 0.04) test sets.

#### 4.2.3 Algorithm Architecture

When the standard ResNet‐50 architecture of the baseline CADe model was replaced with a more novel and presumably more robust architecture, the AUC score for this ViT‐based CADe model was similar to the high‐ and moderate‐quality test sets with 84% (*p* = 0.58) and 78% (*p* = 0.11). On the low‐quality test set, AUC was lower with 64% (*p* = 0.005). AUC differences were larger on the moderate (−6%; *p* = 0.03) and low‐quality (−20% *p* = 0.1) test sets.

Results of individual robustness enhancing methods are summarized in Supporting Information S1: Table 1 and Figure S3.

## 5 Discussion

In this study, we investigated the impact of image quality on the performance of CADe systems for early Barrett's neoplasia. The results reveal a significant vulnerability of CADe systems to realistic image quality variation. The CADe system developed along the lines of most current AI systems displayed a drop of AUC up to −12% and a neoplasia miss rate of 53% when exposed to low‐quality images. This is a significant finding and may be a partial explanation of the lower performance of many AI systems when externally evaluated [5, 6, 7].

In this paper, three robustness‐enhancing methods are proposed to bridge the domain gap between the performance of CADe systems developed using expert‐acquired high‐quality imagery and their performance in community hospitals. When we retrained our conventionally trained CADe system integrating these three methods, it outperformed the conventionally trained system on all test sets. More importantly, on moderate and low‐quality images, the performance loss in terms of AUC and sensitivity was significantly smaller, when compared to the conventionally trained system, indicating robustness against heterogeneity in quality. An example is given in Supporting Information S1: Figure S4.

As a secondary analysis, we focused on each of the three robustness enhancing methods separately. Enhancing the training set with a substantial number of randomly selected video frames significantly improved the robustness of the CADe system. Notably, the CADe version that incorporated video‐based training data as its sole modification outperformed the other two methods in terms of performance loss reduction in suboptimal image quality. Only a relatively small drop in AUC and sensitivity (−11% and −11%, respectively) was observed on the low‐quality test set. This improvement is likely attributable to the inherent diversity in image quality found in video frames, as opposed to the more homogeneous quality of still images. One could argue that the observed effect can be solely attributed to the increased size of the training set, which included a tenfold increase in video frames compared to the conventional CADe system. To account for the potential influence of training set size, we conducted an additional experiment using a video frame dataset matched in size to the original still image training dataset. Even with this matched size, a comparable trend persisted, underscoring that data diversity, rather than sheer dataset size, likely contributed to the observed robustness. This has been described in more detail in the Supporting Information S1: (3 and Figure S5).

Incorporating GastroNet‐5M for domain‐specific pretraining into the CADe system's training regimen mainly affected the absolute performance of the CADe system. AUC scores on the low‐, moderate‐, and high‐quality test sets improved by 5%–12% when compared to the conventionally trained system. The relative performance difference in the lower quality test sets was comparable to the conventional CADe system. Still, the enhanced performance on lower‐quality images, particularly in terms of sensitivity, can be viewed as an indication of the system's increased robustness.

Substituting the ResNet‐50 architecture with a presumably more robust ViT architecture as an isolated robustness‐enhancement method did not yield improvements in the CADe system's absolute performance or its reduction in performance loss. This underperformance could stem from the known dependency of ViTs on large training datasets [23]. Given that our training set comprised only 1011 Barrett's images, it is possible that the dataset was too small to utilize the ViTs full potential. This theory is supported by the observation that the combination of the ViT architecture and domain‐specific pretraining with 5 million images did result in more robust performance across high‐, moderate‐, and low‐quality test sets compared to the CADe systems using just one of these methods (Supporting Information S1: Figure S6).

An up‐to‐this‐point unmentioned robustness‐enhancing method is data augmentation. While data augmentation may appear to be a feasible approach to bridging the gap between expert‐acquired data and data from community centers, it has inherent limitations for this application. Augmentation techniques such as brightness adjustments, addition of motion blur, and contrast modification can replicate certain image artifacts and introduce some level of image quality variability. However, these methods are too limited to fully capture the range of quality issues encountered in real‐world settings. Complex factors, such as mucosal cleaning or esophageal expansion, significantly affect image quality in ways that data augmentation cannot simulate. We conducted preliminary experiments with data augmentations that mimicked typical endoscopic artifacts, including motion blur, focal blur, and varying lighting conditions. However, these augmentations did indeed not result in substantial improvements in performance robustness. Further details on these experiments are provided in the Supporting Information S1: (3–4).

A notable finding in this study was that specificity of the conventionally trained CADe system increased on lower quality images. Although it remains speculative, this could be attributed to the fact that the conventional system is not familiar with low‐quality images that contain e.g., improper illumination or blurriness. As blur and poor illumination are generally no image characteristics of neoplasia, they may be classified as non‐dysplastic. This bias toward negative predictions could be detrimental to the efficacy of the system in daily practice. In contrast, the robust CADe system *has* been trained with blurry and poorly illuminated neoplastic and non‐dysplastic data (i.e., video frames), which lowers the chance of introducing unwanted biases.

This study has some unique features. First, it is one of the few studies addressing the current domain gap between academic development and daily practice, leading to degradation of reported AI performance. Second, we provide multiple solutions to bridge this domain gap, which are relatively easy to implement by other research groups, such as the use of video frames and selecting more appropriate model architectures. As domain‐specific pretraining requires large datasets and vast computational resources, it may be challenging for others to implement. To circumvent this issue, our group is planning to release the GastroNet‐5M dataset for public use. Several GastroNet‐pretrained models are already available [18]. Third, the test sets were carefully designed to comprise a large variety of image quality. The paired nature of the test sets results in a reliable and methodologically solid experiment. Finally, given the close resemblance of the training datasets with those of our recently published CADe system [12], these suggested modifications can be directly integrated into the current algorithm infrastructure.

The study also has some limitations. First, all data in this study originate from expert centers, which may limit the generalizability of our results. While the test set encompassed three carefully curated subsets reflecting diverse endoscopic image quality, these remain a surrogate for community‐based data. Ideally, the test data would originate from various community centers to capture its full heterogeneity, including factors such as endoscopic expertise and sedation type. However, collecting large‐scale non‐expert data remains challenging for Barrett's neoplasia due to its low incidence in these centers. Moving forward, we plan to include data from non‐expert endoscopists in future preclinical studies and to evaluate the system in randomized clinical trials within community surveillance settings. Second, this study only involved a fraction of the training data used for our previously published CADe system [12]. The aim of this study was not to strive for an optimally performing CADe system but to evaluate domain‐gap and robustness as a scientific experiment. Third, this study is limited to one specific endoscope manufacturer and a single specific clinical task, i.e., detection of Barrett's neoplasia. However, it is reasonable that these results will extrapolate to other endoscopy systems and clinical tasks. Finally, this study specifically addresses endoscopist‐dependent factors that influence the successful clinical implementation of endoscopic AI systems. Other endoscopist‐independent factors that may affect CADe performance—such as variations in endoscopy equipment and software settings, as well as differences in neoplasia prevalence between test datasets and routine clinical practice—were not addressed.

In conclusion, this study reveals a significant vulnerability of AI systems to variability in endoscopist‐dependent image quality. This poses a major challenge for successful clinical implementation. For the development of future endoscopic AI systems, robustness to data heterogeneity should be taken into account. We propose several methods to improve the robustness of AI applications. All modifications to algorithm development listed above will be incorporated into an updated version of our previously published system for the detection of early Barrett's neoplasia. Given the adaptability of these changes to other endoscopic deep learning systems, we encourage other groups to explore if these modifications are advantageous for their own applications.

## Conflicts of Interest

JJB reports financial support for IRB approved research from C2Therapeutics, Pentax Medical, Medtronic, Olympus and Aqua Medical. PHW received financial support for IRB approved research from Olympus.

## Supporting information

Supporting Information S1

## Data Availability

Study results and code can be shared upon reasonable request (m.jong3@amsterdamumc.nl). Endoscopic datasets cannot be shared due to IP‐related constraints.
