# Supplementary material for: Challenges in Implementing Endoscopic Artificial Intelligence: The Impact of Real‐World Imaging Conditions on Barrett's Neoplasia Detection
Source: United European Gastroenterol J. 2025 Mar 21;13(6):929–37. doi: 10.1002/ueg2.12760 (PMC12269737; doi:10.1002/ueg2.12760)
Supplement: Supplementary file 1 — Supporting Information S1 [file UEG2-13-929-s001.docx]

**SUPPLEMENTARY MATERIAL**

**BONS-AI Consortium**

The authors wish to thank the members and collaborators of the BONS-AI Consortium, as listed below in alphabetical order:

Alaa Alkhalaf, Isala Hospital, Zwolle, the Netherlands;

Lorenza Alvarez Herrero, St. Antonius Hospital, Utrecht, the Netherlands;

Francisco Baldaque-Silva, Karolinska University Hospital, Stockholm, Sweden;

Maximilien Barret, Cochin Hospital, Paris, France;

Jacques J Bergman, Amsterdam UMC, Amsterdam, the Netherlands;

Torsten Beyna, Evangelisches Krankenhaus Düsseldorf, Düsseldorf, Germany;

Tim G Boers, TU Eindhoven, Eindhoven, the Netherlands;

Lucas C Duits, Amsterdam UMC, Amsterdam, the Netherlands;

Rixta A H, Amsterdam UMC, Amsterdam, the Netherlands;

Peter Elbe, Karolinska University Hospital & Karolinska Institute, Stockholm, Sweden;

Kiki N Fockens, Amsterdam UMC, Amsterdam, the Netherlands;

Albert J de Groof, Amsterdam UMC, Amsterdam, the Netherlands;

Martin H Houben, HagaZiekenhuis, The Hague, the Netherlands;

Martijn R Jong, Amsterdam UMC, Amsterdam, the Netherlands;

Tim J M Jaspers, TU Eindhoven, Eindhoven, the Netherlands;

Jelmer B Jukema, Amsterdam UMC, Amsterdam the Netherlands;

Carolus H. J. Kusters, TU Eindhoven, Eindhoven, the Netherlands;

Rosalie C Mallant-Hent, Flevoziekenhuis, Almere, the Netherlands;

Guiomar Moral Villarejo, Nottingham University Hospitals NHS Trust, Nottingham, United Kingdom

Wouter Nagengast, UMC Groningen, Groningen, the Netherlands;

Jacobo Ortiz Fernández-Sordo, Nottingham University Hospitals NHS Trust, Nottingham, United Kingdom

Oliver Pech, St. John of God Hospital, Regensburg, Germany;

Roos E Pouw, Amsterdam UMC, Amsterdam, the Netherlands;

Joost A van der Putten, TU Eindhoven, Eindhoven, the Netherlands;

Krish Ragunath, Royal Perth Hospital, Perth, Australia;

Pieter Scholten, Onze Lieve Vrouwe Gasthuis, Amsterdam, the Netherlands;

Stefan Seewald, Klinik Hirslanden, Zurich, Switzerland;

Fons van der Sommen, TU Eindhoven, Eindhoven, the Netherlands;

Laureen S Visser, Amsterdam UMC, Amsterdam, the Netherlands;

Jessie Westerhof, UMC Groningen, Groningen, the Netherlands;

Bas L Weusten, UMC Utrecht & St. Antonius Hospital, Utrecht, the Netherlands;

Peter H de With, TU Eindhoven, Eindhoven, the Netherlands;

**Data acquisition and curation**All data used for training the different versions of CADe systems originated from previous studies^1, 2^. Included cases were patients under surveillance for NDBE or undergoing endoscopic treatment of BE neoplasia. Images and videos were prospectively collected in 15 hospitals according to a standardized acquisition protocol. Images and videos were recorded using white light endoscopy. Ten second videos were recorded throughout the complete Barrett’s segment with 2 cm intervals, followed by two still images. All imagery was recorded using Olympus HQ190 and EZ1500 endoscopes and Olympus CV190 and X1 processors. Data was captured using MediCap USB300 (MediCapture, Plymouth Meeting, PA, USA) for CV190 processors and HVO-4000MT (Sony Corporation, Tokyo Japan) for X1 processors. Neoplastic and non-dysplastic cases originated from the same patient population (i.e. patients visiting the hospital) and had similar properties in terms of metadata (e.g. endoscope type, distal cap attachment usage).

The review of images was conducted by three research fellows (KNF, JBJ, and MRJ) to confirm certain criteria. Images of non-dysplastic Barrett’s esophagus were included only if they showed no signs of dysplasia in biopsy samples from the same endoscopy and there was no history of endoscopic treatment for Barrett's. Images in the neoplastic group showed clear lesions with either high-grade dysplasia or adenocarcinoma, confirmed by endoscopic resection at the time. All patients with neoplastic lesions had no previous treatment for Barrett’s neoplasia.

**Algorithm development**

All models undergo training using Binary Cross Entropy as the loss function, employing a learning rate set at 0.0001 for 100 epochs. The model achieving the highest AUC score on the validation set is preserved and subsequently used for the final evaluation on the test sets. For training, images were resized to 256x256 pixels and data augmentation techniques encompass random resize cropping within a scale range of (0.7, 1.1), alongside random horizontal and vertical flipping, along with rotations at 0, 90, 180, and 270 degrees. All models were trained on a single GeForce RTX 3090 Ti GPU (NVIDIA Corp., CA, USA) with a batch size of 128 images.

**Training set size vs. diversity**

In this study, we experimented with the inclusion of large quantities of video frames in the training set to increase image quality diversity. This resulted in improved scores on the high quality test data *and* on reduced performance reduction on lower quality test data. However, these results may be confounded by the increased size of the video frame dataset relative to the still-image dataset, making it unclear whether the observed improvements were due to data diversity or simply the larger training set size. To clarify this, we conducted an additional experiment in which the size of the video frame dataset was matched to the original still-image dataset. This allowed us to isolate the effect of image diversity from that of dataset size. The results showed that the CADe model trained on this matched-size video frame dataset no longer outperformed the conventionally trained CADe system on high-quality test data. However, on lower quality test data, the model trained with this matched-size, video-based dataset continued to exhibit a reduced performance decline compared to the conventionally trained CADe system. These findings suggest that the diversity of image quality—rather than just dataset size—plays a critical role in the model's robustness against lower quality images.

Further details on these results can be found in Supplementary Figure S5.

**Evaluation of Data Augmentation as a Robustness-Enhancing Method**

In addition to the primary robustness-enhancing methods explored in this study, we also investigated data augmentation as a potential approach to increase model robustness against image quality variations. Data augmentation techniques, such as rotating, cropping and contrast adjustment, are often used to introduce variability into training datasets and avoid overfitting of AI systems. Therefore, the inclusion of additional data augmentation techniques may seem like a valid solution for bridging the gap between the high-quality, expert-acquired images used in CADe development and the more variable quality of images encountered in community hospital settings. We performed some preliminary experiments to simulate endoscopy specific image quality variation. Additional data augmentations included focal blur, motion blur, local over/under exposure and specific contrast, sharpness and brightness adjustments. These augmentation did not result in any significant improved AUC scores on high (84%; p=0.19), moderate (82%; p=0.37) or low-quality (65%; p=0.19) test datasets.

While data augmentation can replicate certain image characteristics, it is inherently limited in scope. Specifically, augmentation methods are unable to address more complex, real-world quality issues such as inadequate esophageal cleaning or suboptimal esophageal expansion.

**Supplementary Tables**

**Table S1.** Specifications on the robustness test set.

|  | Neoplastic group  N = 61 | NDBE group  N = 56 |
| --- | --- | --- |
| **Primary Paris type, n (%)** | | |
| 0-Ip/s | 17 (28%) | - |
| 0-II | 40 (66%) | - |
| 0-III | 4 (6%) | - |
| **Pathology**  Worst overall histology, n (%) | | |
| Intestinal metaplasia / No dysplasia | - | 56 (100%) |
| High-grade dysplasia | 22 (36%) | - |
| Esophageal adenocarcinoma | 39 (64%) | - |
| **Endoscope / Processor, n (%)** | | |
| EZ1500 / X1 | 32 (52%) | 23 (41%) |
| HQ190 / CV190 | 29 (48%) | 33 (59%) |

**Table S2.** Results of all CADe versions and performance differences.

| **Test Set** | **Metric** | **Conventional CADe System** | **Robust CADe System** | | **In-Domain Training Data (Video Frames)** | | **Domain-Specific Pretraining** | | **Robust Architecture** | |
| --- | --- | --- | --- | --- | --- | --- | --- | --- | --- | --- |
|  |  | **Score (mean)** | **Score (mean)** | **P-value** | **Score (mean)** | **P-value** | **Score (mean)** | **P-value** | **Score (mean)** | **P-value** |
| High-Quality | AUC | 83% | 92% | 0.0039 | 87% | 0.0503 | 95% | 0.0004 | 84% | 0.5802 |
|  | Sensitivity | 85% | 88% | 0.4415 | 79% | 0.2698 | 85% | 0.8384 | 80% | 0.2479 |
|  | Specificity | 67% | 81% | 0.0025 | 74% | 0.0756 | 90% | 0.0063 | 68% | 0.8164 |
| Moderate-Quality | AUC | 80% | 93% | 0.0006 | 85% | 0.0125 | 89% | 0.0069 | 78% | 0.1116 |
|  | AUC difference | -3% | +1% | 0.0043 | -1% | 0.0421 | -5% | 0.1120 | -6% | 0.0342 |
|  | Sensitivity | 62% | 87% | 0.0108 | 80% | 0.0632 | 84% | 0.0157 | 71% | 0.0959 |
|  | Sensitivity difference | -22% | -1% | 0.0010 | +1% | 0.0010 | -1% | 0.0019 | -9% | 0.0457 |
|  | Specificity | 78% | 84% | 0.0865 | 76% | 0.6750 | 78% | 0.9994 | 65% | 0.0085 |
|  | Specificity difference | +11% | +3% | 0.1031 | +2% | 0.1216 | -12% | 0.0029 | -3% | 0.0805 |
| Low-Quality | AUC | 71% | 85% | 0.0001 | 76% | 0.1727 | 76% | 0.1438 | 64% | 0.0050 |
|  | AUC difference | -12% | -7% | 0.0163 | -11% | 0.6341 | -19% | 0.0365 | -20% | 0.0124 |
|  | Sensitivity | 47% | 78% | 0.0024 | 68% | 0.1148 | 65% | 0.0241 | 56% | 0.0403 |
|  | Sensitivity difference | -37% | -10% | 0.0001 | -11% | 0.0196 | -20% | 0.0092 | -25% | 0.0397 |
|  | Specificity | 83% | 77% | 0.2343 | 70% | 0.0186 | 79% | 0.2604 | 65% | 0.0008 |
|  | Specificity difference | +16% | -4% | 0.0138 | -4% | 0.0148 | -11% | 0.0044 | -3% | 0.0273 |

**Supplementary Figures**


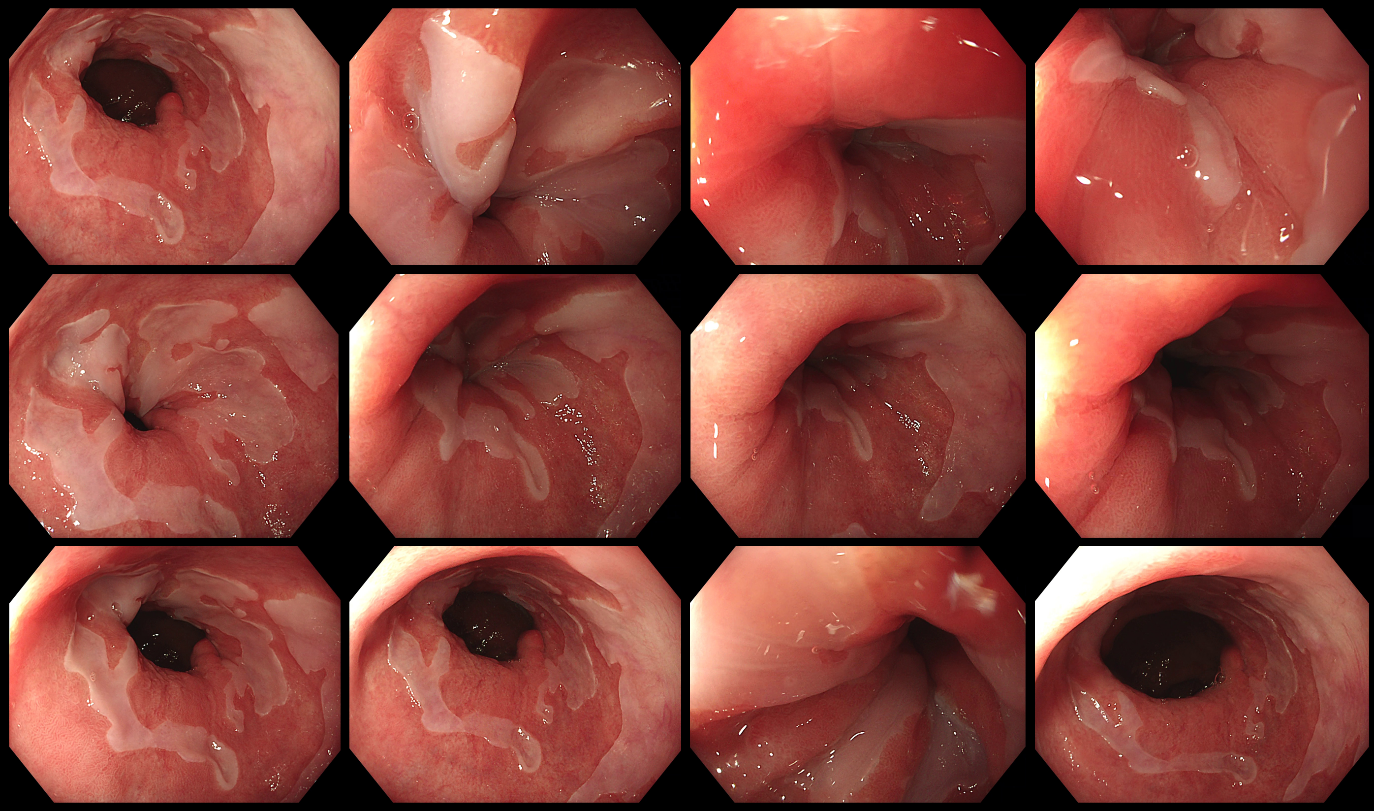


**Figure S1.** Example of randomly selected video frames. The sequences exhibit a broader range of image quality than is typically observed in endoscopic still images.


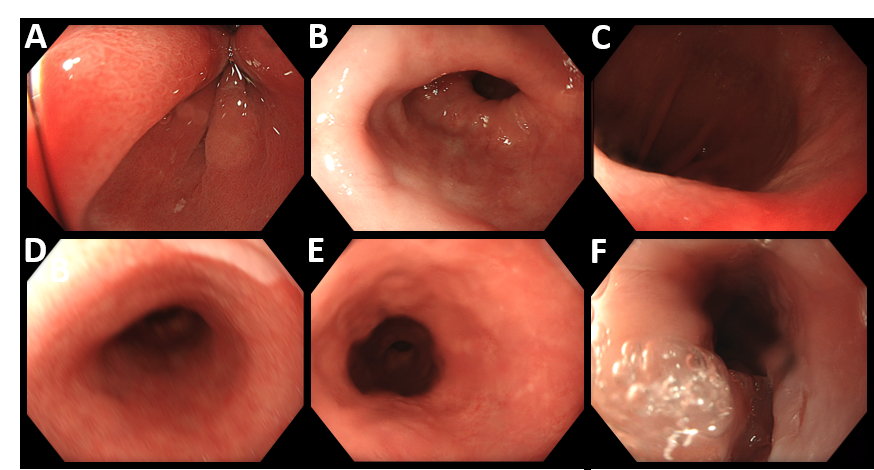


**Figure S2.** Examples of suboptimal images. Each displays a distinct issue: A) Inadequate esophageal expansion; B) Overexposure; C) Underexposure; D) Motion-induced blur; E) Fogged lens; F) Mucus.

**Figure S3.** Results of the CADe systems trained with one individual robustness enhancing method. The dashed bars represent the scores on the high-quality test set.


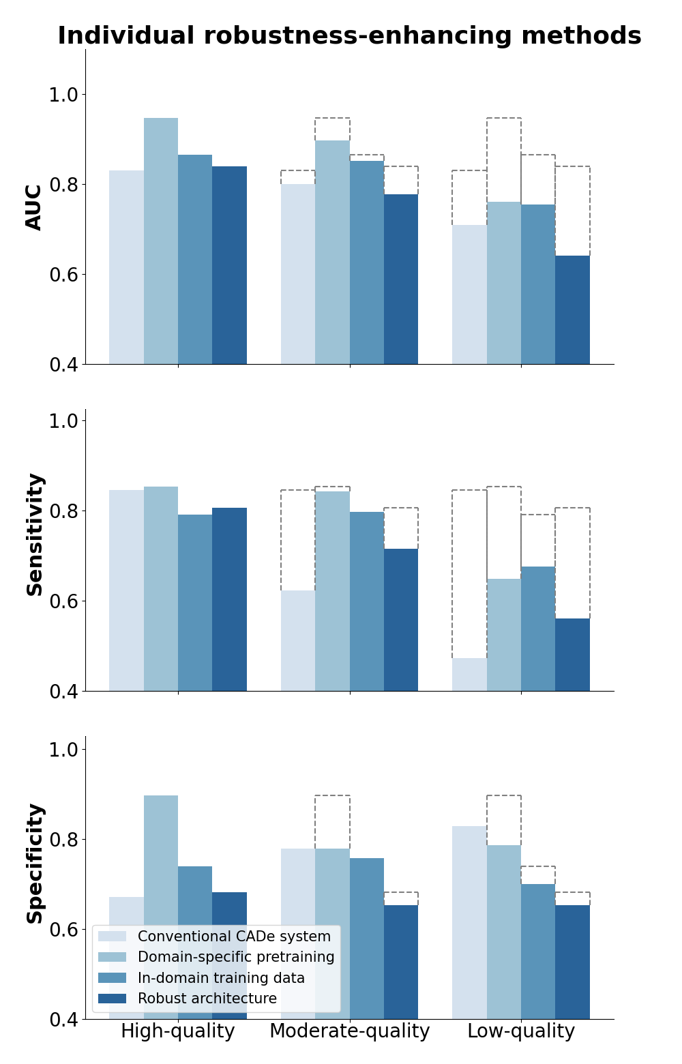


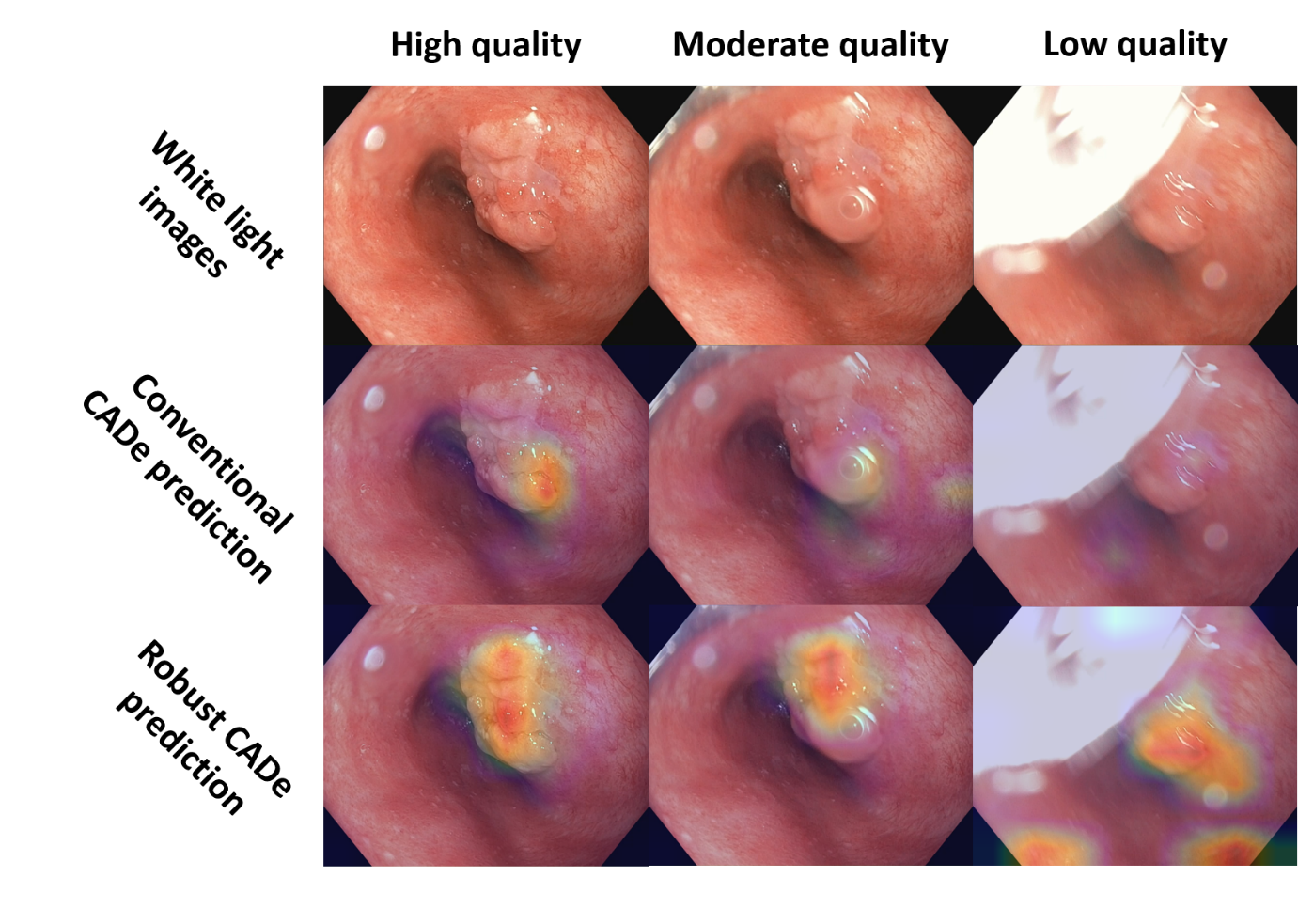


**Figure S4.** Example of test set triplet with a high, moderate and low-quality image. Saliency maps showcase the predictions by the conventionally trained CADe system and the robust CADe system. The latter displays more robust predictions when confronted with lower quality input.


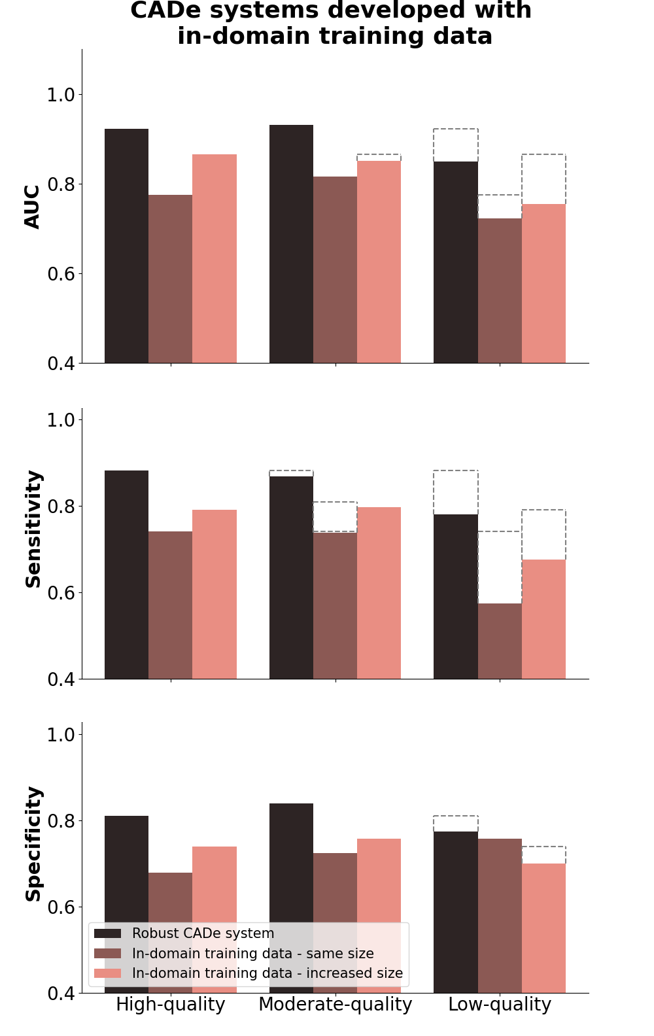


**Figure S5.** Results of the conventional CADe system and the CADe systems trained on video frames. The conventional and ‘same size’ CADe system are trained on equal amount of still images and video frames respectively, while the ‘increased size’ CADe system was trained on a training set 10x the size. The dashed bars represent the scores on the high-quality test set.

**
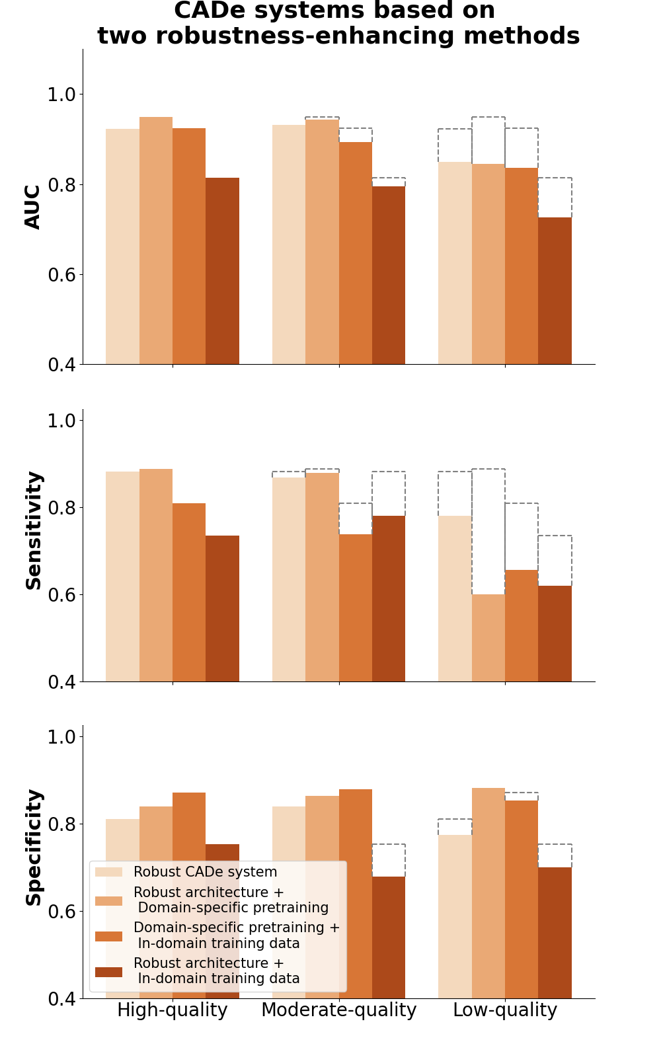
**

**Figure S6.** Results of the robust CADe system and the CADe systems trained with two robustness-enhancing methods. The dashed bars represent the scores on the high-quality test set.

**References**

1. Fockens N, Jong MR, Jukema JB, Pouw RE, Duits LC, Van Munster SN, et al. A deep learning system for detection of early Barrett's neoplasia: a model development and validation study. The Lancet Digit Health. 2023;5:905-21.

2. Fockens KN, Jukema JB, Boers T, Jong MR, van der Putten JA, Pouw RE, et al. Towards a robust and compact deep learning system for primary detection of early Barrett's neoplasia: Initial image-based results of training on a multi-center retrospectively collected data set. United European Gastroenterol J. 2023;11(4):324-36.
